# The Ubiquitin Ligase ASB4 Promotes Trophoblast Differentiation through the Degradation of ID2

**DOI:** 10.1371/journal.pone.0089451

**Published:** 2014-02-21

**Authors:** W. H. Davin Townley-Tilson, Yaxu Wu, James E. Ferguson, Cam Patterson

**Affiliations:** 1 McAllister Heart Institute, University of North Carolina at Chapel Hill, Chapel Hill, North Carolina, United States of America; 2 Department of Cell Biology and Physiology, University of North Carolina at Chapel Hill, Chapel Hill, North Carolina, United States of America; VU University Medical Center, Netherlands

## Abstract

Vascularization of the placenta is a critical developmental process that ensures fetal viability. Although the vascular health of the placenta affects both maternal and fetal well being, relatively little is known about the early stages of placental vascular development. The ubiquitin ligase Ankyrin repeat, SOCS box-containing 4 (ASB4) promotes embryonic stem cell differentiation to vascular lineages and is highly expressed early in placental development. The transcriptional regulator Inhibitor of DNA binding 2 (ID2) negatively regulates vascular differentiation during development and is a target of many ubiquitin ligases. Due to their overlapping spatiotemporal expression pattern in the placenta and contrasting effects on vascular differentiation, we investigated whether ASB4 regulates ID2 through its ligase activity in the placenta and whether this activity mediates vascular differentiation. In mouse placentas, ASB4 expression is restricted to a subset of cells that express both stem cell and endothelial markers. Placentas that lack *Asb4* display immature vascular patterning and retain expression of placental progenitor markers, including ID2 expression. Using JAR placental cells, we determined that ASB4 ubiquitinates and represses ID2 expression in a proteasome-dependent fashion. Expression of ASB4 in JAR cells and primary isolated trophoblast stem cells promotes the expression of differentiation markers. In functional endothelial co-culture assays, JAR cells ectopically expressing ASB4 increased endothelial cell turnover and stabilized endothelial tube formation, both of which are hallmarks of vascular differentiation within the placenta. Co-transfection of a degradation-resistant *Id2* mutant with *Asb4* inhibits both differentiation and functional responses. Lastly, deletion of *Asb4* in mice induces a pathology that phenocopies human pre-eclampsia, including hypertension and proteinuria in late-stage pregnant females. These results indicate that ASB4 mediates vascular differentiation in the placenta via its degradation of ID2.

## Introduction

Vasculogenesis, the formation of new blood vessels from the *de novo* production of endothelial cells, is divided into two categories: extraembryonic (occurring in the yolk sac and allantois) and embryonic (restricted to the embryo itself) [1]. Extraembryonic blood vessel formation precedes embryonic vasculogenesis and provides communication between the fetal circulation and the yolk sac to facilitate the transfer of nutrients and blood gases to the developing embryo [2]. Extraembryonic vasculogenesis supplies the allantois with primitive vessels in preparation for chorion fusion and is responsible for placental development and umbilical vessel formation, thus initiating the vascular connection between the fetal and maternal placental tissues [3]. This vascularization of the early placenta is crucial for the health and viability of not only the fetus, but also the mother [4]–[6]. However, little is known about the key mediators of early placental vascular development.

During human pregnancy, a population of undifferentiated multipotent placental cells, termed cytotrophoblasts (CTBs), differentiate into villous and extravillous trophoblasts that form and remodel the placental vasculature [7]. Villous trophoblasts have endothelial cell functions in the chorionic villi and also fuse into syncytiotrophoblasts [8]. Extravillous trophoblasts invade and migrate through the junctional zone of the placenta into the maternal decidua, where they replace the endothelial cells that line the spiral arteries [5]. These differentiated endothelial-like CTBs cells form tight junctions in the arteries, creating high capacity, low resistance blood vessels that allow for the exchange of blood gasses and nutrients from the mother to the developing fetus [9]. Though not entirely analogous, many of these same processes are conserved in mice [10]. That is, cells originating from a trophoblast (TB) stem cell progenitor migrate and invade the maternal arteries, but are thought to derive from trophoblast giant cell intermediaries, rather than cytotrophoblast lineages [11]. Disruption of the migration and invasion of differentiating TB cells can result in poorly formed vessels that lead to vascular insufficiency of the placenta and fetus, which in turn may result in pre-eclampsia [6]. Pre-eclampsia is characterized as the sudden onset of maternal hypertension, proteinuria, and edema [12]. The underlying pathophysiology of pre-eclampsia is thought to be rooted in vascular dysfunction [13], which may be due to aberrant early TB differentiation [14], though the precise genesis of this disease remains unknown.

Previous work in this laboratory demonstrated that Ankyrin repeat, SOCS box-containing 4 (ASB4) is an oxygen-sensitive E3 ligase that is abundantly expressed in the developing placenta and is highly upregulated during the differentiation of embryonic stem (ES) cells into endothelial cell lineages [15]. In addition, *Asb4* transcription decreases when challenged by laminar shear stress in endothelial cells [16] highlighting the importance of ASB4 in the vasculature. ASB4 is one of 18 proteins in the ASB family, which are part of the suppressors of cytokine signaling (SOCS) super-family. ASB4 contains nine ankyrin repeats, seven of which are highly conserved, and a C-terminal SOCS box [17]. Ankyrin repeats are common 33-residue motifs that mediate protein-protein interactions and are found in proteins with functions ranging from development to transcription and cell cycle control [18]. Like other members of the ASB family [19], ASB4 associates with cullin, elongin, and ROC/Rbx RING-finger proteins (possibly because ASB4 lacks a RING-finger domain), which are all part of the ubiquitin ligase complex [15]. There is little evidence indicating a central function of ASB4. However, areas of high energy consumption (e.g., testes, heart, and brain) in adult mice have ASB4 ligase activity [20]–[22], reinforcing our hypothesis that ASB4 regulates vascular development and differentiation [23], [24]. Taken together, these findings suggest that ASB4 may mediate vascular differentiation through its ligase activity. However, few putative ASB4 substrates or *in vivo* functions are known.

Based on the high expression levels of *Asb4* in the developing placenta, coinciding with the role of ASB4 in vascular differentiation [15], we reasoned that any putative substrates would share expression patterns and function within in the developing vasculature. Inhibitor of DNA binding 2 (ID2) is a tightly regulated mediator of placental development and vascular differentiation [25]–[27]. Further, ID2 is involved in other vascular events, including angiogenesis [26] and tumor cell migration and invasion [28]. ID2 is part of the anti-differentiation ID protein family, which shares significant structural similarity to the basic-helix-loop-helix (bHLH) family of proteins but lacks the basic domain [29]. ID proteins block the transcription of pro-differentiation elements by preventing bHLH dimerization and consequent translocation into the nucleus [30]. Due to the spatial and temporal overlap and the functional contrast between these two proteins, we hypothesized that ASB4 negatively regulates placental endothelial differentiation via and degradation of ID2. We also hypothesized that ASB4’s inhibition of ID2 would have a net pro-differentiation effect, and that loss of *Asb4* would impair vascular differentiation within the developing placenta.

In this report, we investigated the role of ASB4 in TB cell differentiation and function and identified ID2 as a substrate of ASB4s ubiquitin ligase activity. Placentas isolated from *Asb4^−/−^* mice exhibited vascular differentiation defects, dysmorphic placental vessels, vascular dysfunction, and spontaneous abortion in a subset of fetuses. Using cell culture models, we found that ASB4 directly interacted with ID2, leading to ID2’s ubiquitination and subsequent degradation in JAR cells. Further, ASB4 promoted aspects of placental cell differentiation and endothelial cell replacement and vessel stability. Co-transfecting *Asb4* with *Id2* mutants that are resistant to proteasomal degradation abolished these effects. Lastly, pregnant *Asb4^−/−^* mice exhibited symptoms consistent with pre-eclampsia, including proteinuria and hypertension. Together, these findings indicate that ASB4 regulates TB cell differentiation into placental vasculature through the degradation of ID2 and that loss of *Asb4* in the developing placenta contributes to placental disease.

## Methods

### Mouse Generation, Blood Pressure, and Proteinuria

The *Asb4^−/−^* mouse generation is described by Ferguson [31]. Briefly, exon 1 of *Asb4* was flanked by loxP excision sites in the pAMC vector. Positive recombinants were electroporated into 129 SvEv ES cells and cultured with appropriate selection enzymes. ES cells were then injected into C57Bl/6 blastocysts and implanted into pseudopregnant females. The resultant chimera (*Asb4^flox/+^*) was then mated with EIIa-cre mice to excise the loxP sites. These mice were further bred to 129 SvEv wild-type mice to ensure germ-line transmission of the deletion and to outbreed the cre allele, generating *Asb4^+/−^* mice on the 129 SvEv background.

Maternal blood pressure was measured in conscious, pregnant mice at the gestational stage indicated using a CODA 8 tail-cuff monitor (Kent Scientific, Torrington, CT, USA). Mice were habituated to the machine for one day prior to data collection and assayed for five consecutive days. Urinary creatinine and albumin protein levels were measured using the Creatinine Companion and Albuwell M Test kits, respectively (Exocell, Philadelphia, PA, USA). Urine collection consisted of placing isolated mice in metabolic cages (a generous gift from Dr. Nobuyo Maeda [University of North Carolina]) for 24 hours. Food and water were provided ad libitum, and urine was collected in a microcentrifuge tube placed below the mesh flooring. Particulate matters and solids were removed from the samples by benchtop centrifugation, and urine was stored at −20°C until assayed. Placental disc invasion was assessed in E17.5 placentas as described in Dokras et al. [32]. All experiments were approved by the Institutional Animal Care and Use Committee of the University of North Carolina at Chapel Hill.

### In situ Hybridization, Immunofluorescence, and Immunohistochemistry

In situ hybridization for *Asb4* was performed by the UNC In Situ Hybridization Core Facility on 16-µm thick cryosections of placental tissue harvested under RNase-free conditions from E11.5 wild-type mice. To generate the *Asb4* probe, a ∼900 bp fragment of *Asb4* was TA-cloned into pCRII-TOPO using the primers 5′-CTCCGAGGATGGACGGCATCACTGCCCCTATC-3′ and 5′-CTCAGGCTGTGCAGCAGGACGC-3′. The fragment was excised using NotI and BamHI restriction enzymes. Sense and anti-sense probes were generated by transcription with T7 and Sp6 polymerase, respectively. Probes were digoxigenin-labeled prior to hybridization.

Immunofluorescence and immunohistochemistry were performed as described in Waldo et al. [33] on placental tissue sections at the indicated embryonic day. Briefly, tissue was harvested and either flash frozen or fixed in 4% paraformaldehyde overnight with subsequent cryoprotection in 30% sucrose. Samples were embedded in OTC Compound (Sakura Finetek, Torrance, CA, USA) and sectioned into 6-µm thick slices by the UNC Histology Research Core Facility. Primary antibodies are as follows: antibody recognizing mouse ASB4 was generated as in Ferguson [31]; c-kit (Cell Signaling Technology, Danvers, MA, USA); PECAM (Becton-Dickinson, San Jose, CA, USA); cytokeratin-17, integrin alpha V, and integrin beta 4 (Abcam); ID2 (Cell Signalling Technology); Von Willebrand factor (Dako, Carpinteria, CA, USA); FITC-conjugated Dolichos biflorus agglutinin (DBA) (Sigma-Aldrich, St. Louis, MO, USA); and phospho-histone 3 (Millipore, Billerica, MA, USA). Alexa Fluor antibodies (Invitrogen, Grand Island, NY, USA) and ABC Elite kits and diaminobenzidine (Vector Labs, Burlingame, CA, USA) were used to detect primary antibodies. Apoptosis was quantified using the ApopTag In Situ Apoptosis detection kit (Millipore). Hematoxylin and eosin staining was performed on fixed frozen sections by the UNC Histology Core Facility. Tissues and cells were imaged on a Nikon E800 upright fluorescent microscope, and ImageJ (http://rsbweb.nih.gov/ij/) was used for quantification and intensity measurements.

### Cell Culture and Immunoblotting

JAR choriocarcinoma cells were obtained from ATCC (Manassas, VA, USA) and maintained in MEM supplemented with 10% FBS. HEK-293T/17 cells and 2H-11 endothelial cells were maintained in DMEM supplemented with 10% FBS. 2H-11 cells were conditioned to constitutively ectopically express ASB4 by transfecting cells with p3xFLAG-CMV10-*Asb4*, and stable clones were selected with G418 for 12 days. Transgene expression was confirmed by anti-FLAG immunoblotting. A stable *Asb4* knockdown cell line was created in 2H-11 cells using mouse *Asb4* shRNA lentiviral particles. Endogenous *Asb4* expression was screened by reverse transcription-PCR. Transfection reactions were performed using LTX and Plus reagent (Invitrogen) according to Wolfe [34]. pCMV2B-*Asb4* and p3xFLAG-CMV10-*Asb4* were generated and used as described in Ferguson [31]. *Id2*-Sport6 and pCS2-*Id2* mutants were generous gifts from Dr. Aaron Ciechanover (Technion-Israel Institute of Technology). siRNA transfections were performed using X-tremeGENE siRNA transfection reagent (Roche, Indianapolis, IN, USA) according to the manufacturer’s instructions. The *siAsb4* duplex sequence is as follows: 5′-CCACAAUGCUACCAUCAA-3′ and 5′-AGUUGAUGGUAGCAUUG-3′, and siRNA was synthesized and duplexed by Integrated DNA Technologies (Coralville, IA, USA). Cell lysis reactions were performed in cell lysis buffer (50 mM Tris, pH 7.5, 150 mM NaCl, 1 mM EDTA, 1 mM EGTA) containing 1% Triton (immunoblots) or 0.5% NP-40 (immunoprecipitations). Cell fractionation assays were performed using NE-PER Nuclear and Cytoplasmic extraction kit (Thermo Scientific, Rockford, IL, USA) according to the manufacturer’s protocol. Cycloheximide was used at 50 µM in DMSO. Immunoprecipitation reactions were lysed as described above and crosslinked with 2 mM DSP (dithiobis[succinimidylpropionate]) (Thermo Scientific) for 2 hours at 4°C. Lysates were pre-cleared with the appropriate species IgG and Protein A/G beads (Santa Cruz Biotechnology) for 1 hour at 4°C and then incubated with either anti-c-myc or anti-FLAG affinity gel (Sigma-Aldrich). Primary antibodies include ID2 (Cell Signaling Technology); FLAG-HRP, myc-HRP, and GAPDH (Sigma-Aldrich); KDM1 and MEK1 (Abcam, Cambridge, MA, USA); and HA-HRP (Roche). Proteins were detected using HRP-conjugated, species-appropriate secondary antibodies (Sigma-Aldrich) and developed using TMA-6 reagents (Lumigen, Southfield, MA, USA).

### In vitro Ubiquitination Assay

Recombinant ID2 was generated using an ID2-GST fusion construct (pGEX-2T-*Id2*) graciously supplied by Dr. Antonio Iavarone (Columbia University) in BL21(DE3)pLysS chemicompetent cells (Agilent Technologies, Santa Clara, CA, USA). Recombinant ASB4 was generated by ectopically expressing p3xFLAG-CMV10-*Asb4* in HEK-293T/17 cells. E1 (Ube1), E2 (UbcH5a), ATP, and ubiquitin were purchased from Boston Biochem (Cambridge, MA, USA). The reaction buffer was as follows: 50 nM E1, 2.5 µM E2, 2.5 µM ASB4, 5 µM ID2, 2.5 mM ATP, 50 mM Tris, pH 7.5, 50 mM KCl, 0.2 mM DTT, and 250 µM ubiquitin. Reactions were performed at 37°C for 1 hour.

### Placental Cell Differentiation Assays

Trophoblast stem cells (TBSCs) were isolated as previously described [35] from E7.5 embryos. Cells were grown for 6–8 weeks under normal culture conditions to minimize spontaneous differentiation. Cells were cultured for 72 hours without serum and then visualized for the presence of trophoblast giant cells, a hallmark of TBSC differentiation in culture. JAR cells were induced to secrete human chorionic gonadotropin (hCG) using N(6),2'-O-dibutyryladenosine 3′:5′ cyclic monophosphate (dbcAMP, Sigma-Aldrich) at 1 mM for 24 hours as described in Hohn et al. [36]. hCG was measured using an ELISA (DRG International, Mountainside, NJ, USA), and concentrations were normalized to cell number. The JAR cell-mediated apoptosis of 2H-11 endothelial cells was evaluated based on Chen et al. [37], using the TUNEL-based ApopTag staining kit (Millipore) as an index of apoptosis. To assay endothelial network stability mediated by placental cells, we adapted the JAR cell/endothelial tube association assay from Aldo et al. [38], using 2H-11 endothelial cells as our vascular network substrate. JAR cells were transfected as indicated, then plated upon 2H-11 cells that had formed tube-like networks. Total area of the JAR/2H-11 network was measured using ImageJ and quantified.

Unless otherwise noted, statistical analysis for all quantification was performed using a two-tailed, unpaired Student’s t-Test. p values are reported in the respective figure legends.

## Results

### ASB4 is Expressed in Undifferentiated TB Cells and is Required for Placental Differentiation

**Figure 1:**
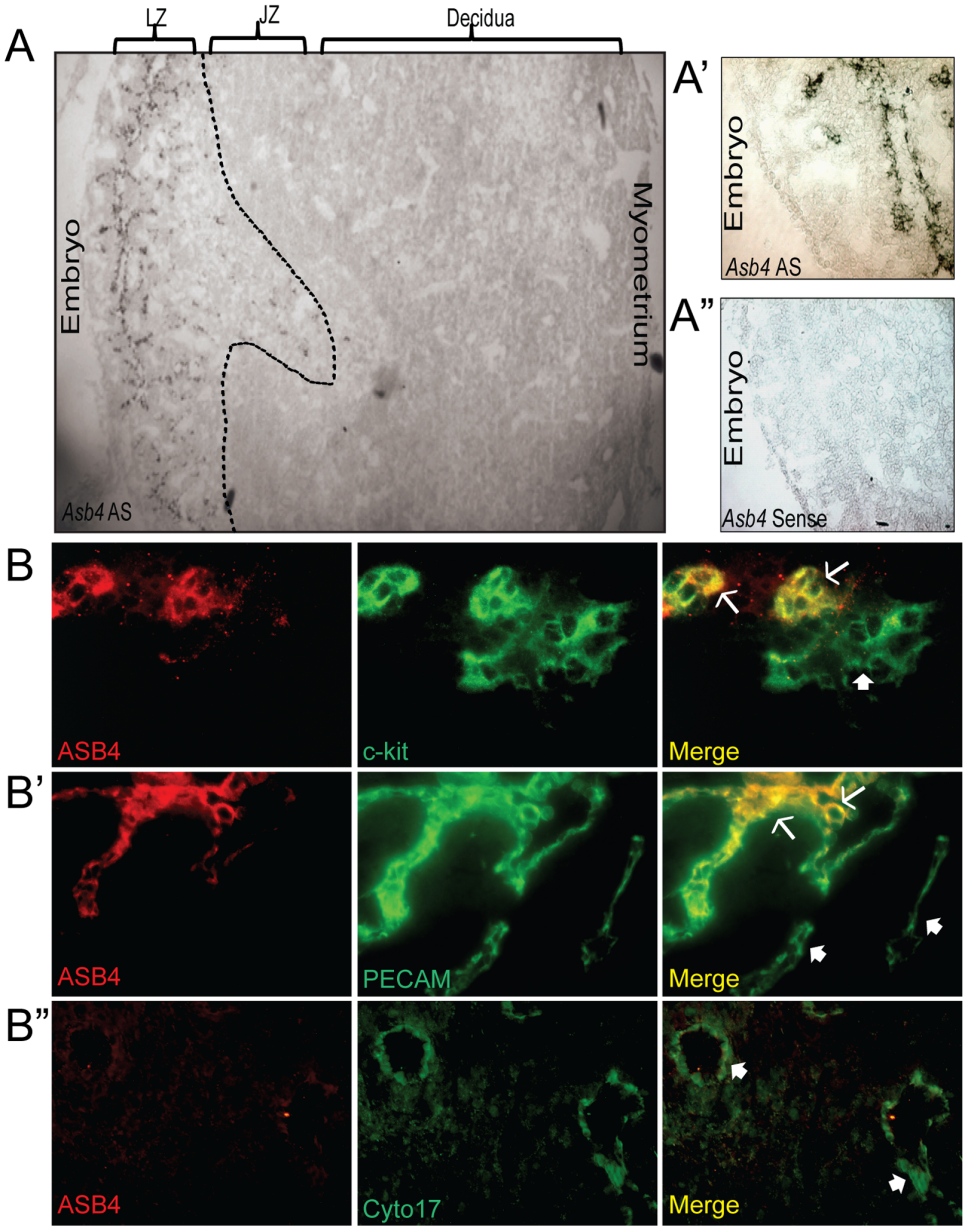
ASB4 is expressed in the developing placental vasculature. A) *Asb4* mRNA is expressed only in the labyrinth zone of developing placentas. *In situ* hybridization was performed on E11.5 placental sections and imaged with bright field microscopy. Wide-field (4x, A) and higher magnification (20x, A’) anti-sense (AS)-probed sections illustrate *Asb4* localized to the labyrinth zone. A sense probe was used as a negative control (A”). B) ASB4 is expressed in a subset of c-kit-positive and PECAM-positive cells but not mature cytokeratin 17-expressing cells. E11.5 placental sections were probed with markers of stem cells (c-kit, B), endothelial cells (PECAM, B’), and differentiated TB cells (cytokeratin 17, B”) and fluorescently imaged at 20x magnification. These images were then merged to show co-localization. ASB4 only co-localizes with cells expressing c-kit and PECAM (arrows) but not cytokeratin 17 (filled arrows). There are also subsets of c-kit or PECAM positive cells that ASB4 did not co-localize at this stage (filled arrows).

*Asb4* is localized to areas of high vascular activity and is highly expressed in the developing placenta [15], [31]. ID2 is also critical in the early development of the placenta, including the maturation of the placental vasculature [26]. Therefore, we hypothesized that ASB4 would be an important modulator of differentiation in the placental vasculature. As shown in Figure 1A (4x magnification) and 1A’ (20x magnification), *Asb4* mRNA was only expressed in the labyrinth zone of E11.5 placentas. This zone exhibits high vascular activity [39] and contains the reservoir of TB cells that cross the junctional zone into the maternal decidua as they mature into functional endothelial-like cells [40], [41]. This observation supports our hypothesis that ASB4 is involved in early vascular differentiation events in the placenta.

To test whether ASB4 is expressed in differentiating cells committing to a vascular lineage, we examined placentas at E11.5, when stem cells undergo both self-renewal and differentiation and the cells adopting a vascular lineage migrate from the stem cell niche [42]. Wild-type placental tissue sections were co-labeled for ASB4 and markers of various stages of TB-to-endothelial differentiation. ASB4 co-localized with markers of pluripotent, cells, specifically in a subset of cells expressing the general stem cell marker c-kit (Figure 1B) [43]. At this stage in mouse placental development, invading and differentiating trophoblasts express c-kit, but terminally differentiated trophoblast giant cells and spongiotrohoplasts do not. Further, c-kit and its ligand SCF are implicated have been implicated as being required for trophoblast spreading and implantation, due to their role in trophoblast differentiation [44]. In addition, ASB4 also co-localized with a subset of PECAM-positive cells (Figure 1B’), suggesting that ASB4 is involved with cells that are differentiating into vascular lineages at this time point. Further supporting a specific role in early vascularization, ASB4 did not co-localize with cytokeratin 17, which is a marker of mature placental endothelial-like cells (Figure 1B”).

**Figure 2:**
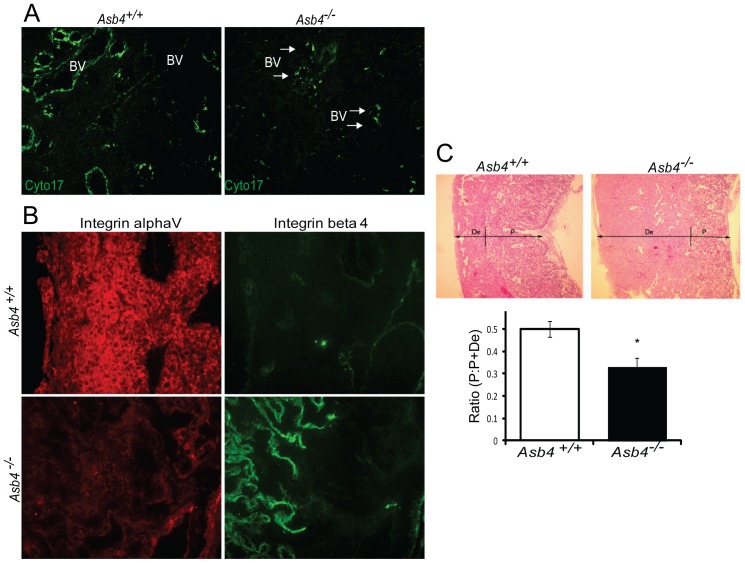
*Asb4^−/−^* placentas express markers of undifferentiated vasculature and TB cells. A) Placentas lacking *Asb4* have reduced cytokeratin 17 expression in near-term placentas. E17.5 placental sections from wild-type and *Asb4^−/−^* mice were labeled with cytokeratin 17 (cyto17), a marker of terminally differentiated endothelial-like TB cells. Blood vessels (BVs) in *Asb4^−/−^* placentas display reduced cytokeratin 17 labeling compared with BVs in wild-type placentas. B) Placentas from E15.5 wild-type and *Asb4^−/−^* placentas were labeled for integrin alpha V, a marker of mature, terminally differentiated TB cells and integrin beta 4, a marker of immature, undifferentiated TB cells. Wild-type placentas express alpha V but not beta 4 integins. Cells in *Asb4^−/−^* placentas retain integrin beta 4 expression and fail to express integrin alpha V. C) Placental disc invasion is reduced in *Asb4^−/−^* mothers at E17.5, indicating restricted trophoblast expansion. The ratio of the placental disc (P) to the total placenta (the sum of the decidua (De) and the placental disc) is decreased in *Asb4^−/−^* placentas compared to wild-type placentas, indicating a defect in TB cell invasion and migration.

Because ASB4 co-localized with early markers of the endothelium, we hypothesized that *Asb4* deletion would lead to functional consequences later in development. Specifically, if ASB4 promoted TB-to-endothelial cell differentiation, then placentas of *Asb4^−/−^* mice should have less mature endothelium than those of wild-type mice. Placentas from wild-type and *Asb4^−/−^* mice were examined at E17.5 and labeled for cytokeratin 17 (Cyto17) to visualize differentiated, mature endothelial-like cells. In wild-type mice, there abundant Cyto17 expression in the lining of the vessels, while in *Asb4^−/−^* mouse placentas there were fewer mature Cyto17-positive cells (Figure 2A), and this decrease was not due to impaired proliferation or increased apoptosis (Figure S1). During normal gestation, placental cells undergoing TB-to-endothelial differentiation undergo integrin “switching” [45], in which undifferentiated TB cells that adopt a vascular phenotype express beta 4 integrins. Later in gestation, when blood vessels have differentiated, beta 4 integrins are turned off, and alpha V integrin is highly expressed [42], [46], consistent with what we see in our wild-type mice (Figure 2B, top panels). *Asb4^−/−^* placentas failed to express integrin alpha V, but maintained beta 4 integrin expression late in gestation (Figure 2B, bottom panels), consistent with a failure of the placenta to undergo integrin switching, indicating an immature and undifferentiated placenta. Placental disc invasion, during which the fetal components of the placenta extend and expand into the maternal decidual layers, was more shallow in *Asb4^−/−^* placentas compared with wild-type mice (Figure 2C) further confirming that placental development is compromised in *Asb4^−/−^* mice in a manner that is consistent with abnormal differentiation [32]. Additional observations indicate that the vasculature expressed markers of injury and dysfunction in near-term (E17.5) *Asb4^−/−^* placentas (Figure S2A) and were mislocalized within the junctional zone rather than the stage-appropriate outer deciduas (Figures S2B and S2C). These results indicate that differentiation defects in *Asb4^−/−^* placentas may have deleterious effects that are observed into late gestation.

**Figure 3:**
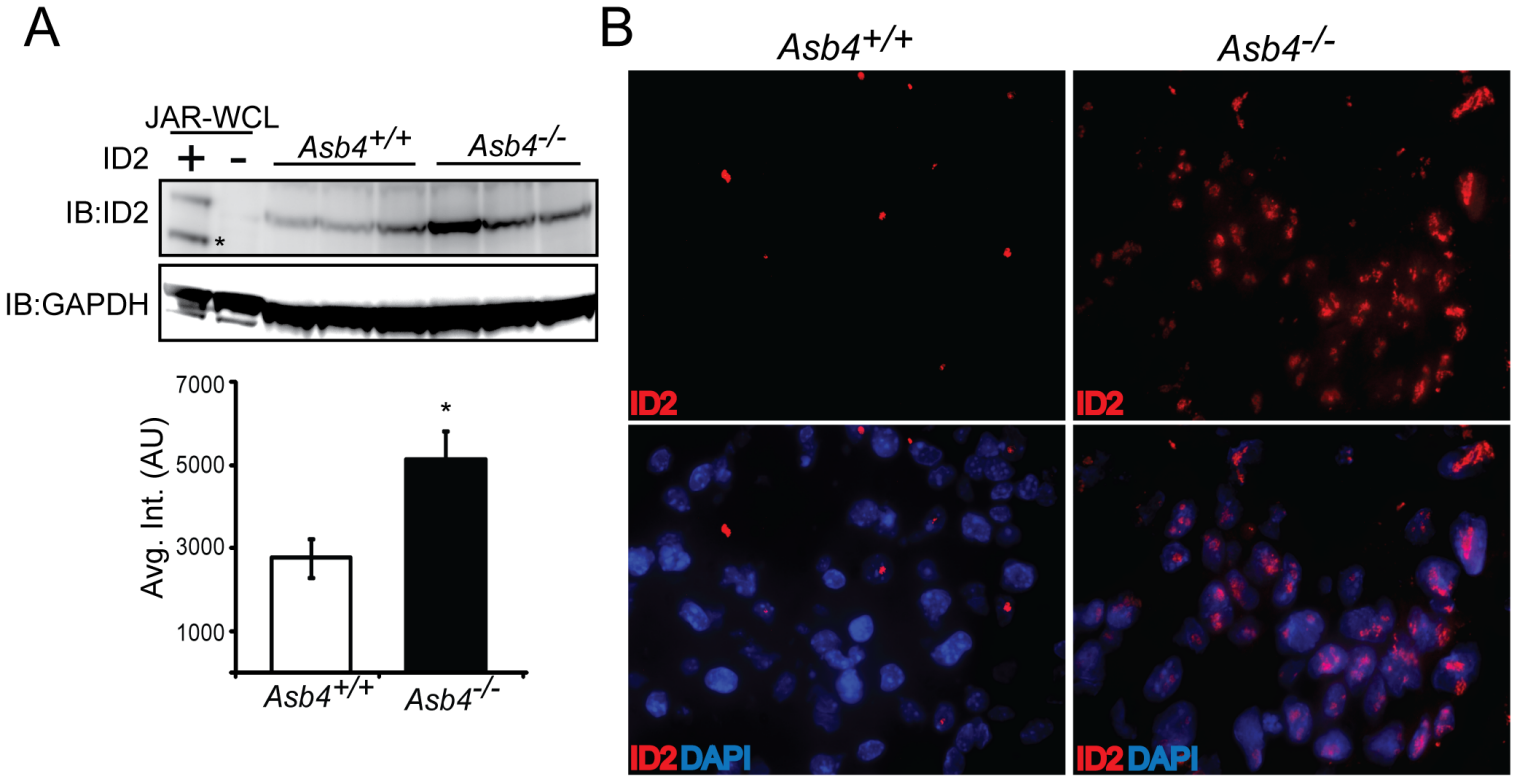
ID2 expression increases in placentas that lack *Asb4*. A) Lysates from three E13.5 wild-type and *Asb4^−/−^* placentas were immunoblotted against ID2 (top panel, asterisk denotes nonspecific band) and quantified (bottom panel). p<0.01. JAR-WCL =  whole cell lysates transfected with *Id2* or vector and run as a positive immunoblotting control. B) E12.5 sections from wild-type and *Asb4^−/−^* placentas were labeled for ID2 (left panel), confirming that wild-type TB cells at this stage have low ID2 expression while ID2 expression is dramatically greater in *Asb4^−/−^* placentas. 100x magnification.

Because *Asb4^−/−^* placentas showed signs of early differentiation defects and impaired vascularization (Figures 2A, B), we examined ID2 expression due to its anti-differentiation role in the placenta [25]. We hypothesized that ID2 expression would be increased in the *Asb4^−/−^* placenta due to ID2’s anti-differentiation role in the placenta, and thus might underlie the observed differentiation defects in *Asb4^−/−^* placentas. In wild-type mice, ID2 expression is downregulated as TB cells differentiate. However, in whole placental cell lysates at E12.5, placentas from *Asb4^−/−^* mice have a ∼2 fold increase in ID2 expression compared with placentas from wild-type mice (Figure 3A), and this finding is confirmed by immunofluorescence in E13.5 placentas (Figure 3B). These data indicate that a subset of TB cell remain undifferentiated in *Asb4^−/−^* placentas, and provide evidence that ASB4 may mediate ID2 expression in the placenta.

### ASB4 Negatively Regulates ID2 Expression through Polyubiquitination and Proteasome Dependant Degradation

**Figure 4:**
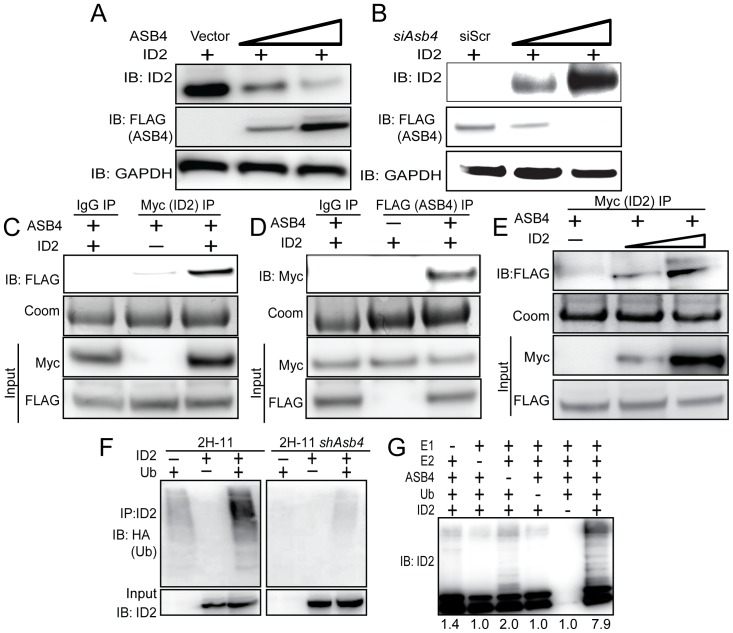
ASB4 negatively regulates ID2 expression through polyubiquitination and associates with ID2 in JAR cells. A) ASB4 represses ID2 expression in a dose-dependent manner. Wild-type *Id2* and vector, 0.5, or 2 µg of *Asb4* were co-transfected in JAR cells. ID2 expression decreases as the ASB4 expression increases. B) ID2 expression increases as ASB4 expression decreases. 2H-11 cells that constitutively express high levels of ectopic ASB4 were transfected with *Id2* and either a scrambled nucleotide siRNA duplex (siScr) or increasing doses (0.15 nM, 0.5 nM) of *siAsb4* duplex. As ASB4 expression decreases, ID2 expression concurrently increases. C and D) ID2 and ASB4 associate in JAR cells. 3xFLAG-tagged *Asb4* and 6xmyc-tagged *Id2* were co-transfected in JAR cells. Lysates were pre-cleared with species-specific IgG and Protein A/G agarose beads were run with these reactions as a control against non-specific binding. Pre-cleared lysates were either immunoprecipitated with anti-myc- or anti-FLAG -conjugated agarose beads and blotted for FLAG or myc, respectively (C, D). Gels were stained with coomassie post-transfer as a loading control for immunoprecipitations. Input represents 2.5% of total lysate. ASB4 is detected in ID2 immunoprecipitation; conversely, ID2 is detected with ASB4 immunoprecipitation. E) 2H-11 cells that stably express FLAG-tagged *Asb4* were transfected with increasing amounts of myc-tagged *Id2*. Cells were lysed and pre-cleared as in C and D, then immunoprecipitated with anti-myc conjugated agarose beads and then blotted for FLAG. FLAG expression increases in parallel with myc expression, indicating specific interaction between ID2 and ASB4. F) ID2 ubiquitination increases in cells with ASB4 expression. Wild-type *Id2* and HA-tagged ubiquitin were transfected into either 2H-11 cells that express endogenous *Asb4* or 2H-11 cells that have *Asb4* constitutively knocked down. ID2 was immunoprecipitated using anti-ID2 and then blotted against HA. Reactions were blotted on the same membrane. Input represents 2.5% of total lysate. Ubiquitination of ID2 increases in endothelial cells that express ASB4 compared with cells that do not. G) ASB4 directly ubiquitinates ID2 *in vitro*. Recombinant ID2 was incubated with recombinant ASB4, and components of the reaction as indicated. Reactions were resolved on SDS-PAGE gels and immunoblotted against ID2. ID2 is ubiquitinated approximately four-fold more with ASB4 than without (lane 3). Quantification of ubiquitination is fold change relative to lane 5 (without ID2).

Given our observation that ID2 was significantly upregulated in *Asb4^−/−^* placentas, we hypothesized that ID2 may be a substrate of ASB4s ubiquitin ligase activity. To test this hypothesis, wild-type *Id2* was co-transfected with *Asb4* in JAR cells, and ID2 expression was examined. JAR cells are a desirable cell type because they do not express endogenous ID2 or ASB4, allowing us to modulate both proteins without endogenous protein interference. Because ID2 is rapidly turned over by myriad other proteins, resulting in a very short half-life (Figure S4B and [47]), we added a low dose of MG-132 to experiments in Figure 4A-E to slow proteasomal degradation events and visualize ID2 protein expression. ASB4 degraded ID2 in a dose-dependent manner in *Asb4* and *Id2* co-transfected JAR cells (Figure 4A). To test whether ID2 expression increased in the absence of ASB4, we transfected *Id2* into 2H-11 endothelial cells that stably overexpressed *Asb4*. ID2 expression increased when co-transfected with increasing amounts of an siRNA duplex targeting *Asb4* (*siAsb4*) (Figure 4B). To determine whether ASB4 binds directly to ID2, we performed co-immunoprecipitation assays using co-transfected 3x-FLAG-tagged *Asb4* and 6x-myc-tagged *Id2* in JAR cells. As shown in Figure 4C, ASB4 was detected when ID2 was immunoprecipitated and, conversely, ID2 was detected when ASB4 was immunoprecipitated (Figure 4D). Further, we observed a dose response of the ID2-ASB4 interaction in 2H-11 cells that had increasing amounts of *Id2* transfected in (Figure 4E). Together, these data show that in placental cells, ASB4 can mediate ID2 expression and that ASB4 and ID2 interact with each other.

Because ASB4 is an E3 ligase, we hypothesized that ASB4 regulates ID2 protein levels by polyubiquitinating ID2 and targeting it for proteasomal degradation. To test this hypothesis, we first co-transfected HA-tagged ubiquitin and myc-tagged *Id2* in 2H-11 cells that either express endogenous *Asb4* or have constitutively knocked down *Asb4* expression to levels undetectable at either the transcript or protein level. We immunoprecipitated ID2 and blotted for HA, expecting a ubiquitin “smear” if ID2 was modified by polyubiquitination. As shown in Figure 4F, ID2 ubiquitination increased dramatically in cells that expressed *Asb4*, compared to cells that do not express *Asb4*. We then tested whether ASB4 could directly ubiquitinate ID2 by performing an *in vitro* ubiquitination assay. We combined recombinant ID2 with ASB4 and the minimal components required for ubiquitination and saw that ASB4 ubiquitinated ID2 four-fold more than the reaction absent of ASB4 (Figure 4G, lane 6 versus lane 3). Importantly, these reactions were performed in the absence of Roc1/Rbx1, the RING-finger protein that associates with ASB4, indicating that ASB4 does not require a RING-finger protein for ubiquitination. Previous reports into the mechanism of ID2 ubiquitination have demonstrated that ID2 is only susceptible to N-terminal ubiquitination [47]. To determine whether ID2’s N-terminus is sensitive to ASB4-mediated degradation, we co-expressed ASB4 with ID2 mutants that lack all lysine residues (LL-ID2) or have 6x-myc tags on either the N-terminus (degradation resistant, DR-ID2) or C-terminus (degradation sensitive, DS-ID2) in JAR cells. Only the N-terminally tagged ID2 (DR-ID2) expression level remained unchanged in the presence of ASB4 (Figure S3), indicating that ASB4 mediates ID2 degradation via N-terminal ubiquitination.

To confirm our hypothesis that ASB4 degrades ID2 in a proteasome-dependent manner, we completely abolished proteasomal activity with a high dose of MG-132 to cells ectopically expressing ID2 and either ASB4 or vector control and compared ID2 expression to cells that were not treated with MG-132. In cells treated with DMSO, ASB4 expression led to reduced expression of ID2 as in Figure 4A. While a high dose of MG-132 slightly increased total ID2 expression in the absence of ASB4, this increase was not diminished by the co-expression of ASB4 in the presence of MG-132 indicating that ID2 is degraded via the proteasome (Figure S4A). Further, when cells ectopically expressing ASB4 and ID2 were treated with cycloheximide to block protein translation, the half-life of ID2 decreased in the presence of ASB4 (Figure S4B). To ensure that the reduction in ID2 expression in the soluble fraction assayed above was not caused by ASB4 inducing ID2 translocation to an insoluble part of the cell, we performed a cell fractionation assay (Figure S4C). There was no observable accumulation of ID2 in any of the cell fractions when co-expressed with ASB4, suggesting that ID2 is not translocated to other insoluble fractions of the cell upon treatment with ASB4.

### ASB4 Mediates Placental Cell Differentiation and Function in vitro

**Figure 5:**
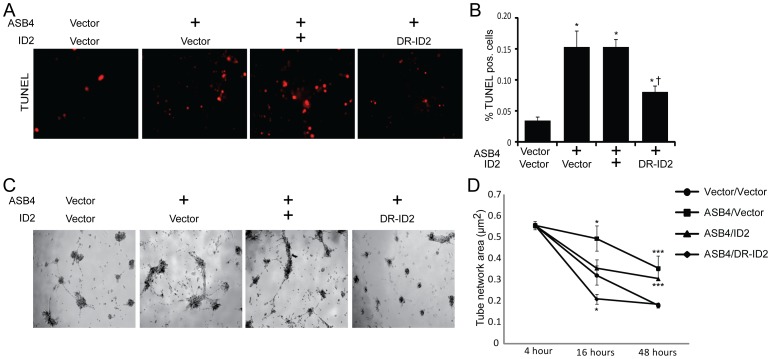
ASB4 promotes JAR cell-mediated endothelial apoptosis and stabilization of endothelial cell networks. A) JAR cells expressing ASB4 promote 2H-11 cell apoptosis. JAR cells were transfected with vector, *Asb4*, *Asb4* and wild-type *Id2*, or *Asb4* and DR-*Id2* prior to being seeded on top of 2H-11 monolayers. TUNEL-positive cells were counted and are presented as the percent of total endothelial cells within the field in panel B. *Asb4*-transfected cells increase apoptosis of the underlying endothelial cells, even when transfected with wild-type *Id2*. DR-*Id2* co-transfected with *Asb4* inhibits JAR-mediated 2H-11 apoptosis. * p<0.01 as compared to vector/vector. † p<0.01 compared to *Asb4*-only transfection. C) JAR cells transfected with *Asb4* promote endothelial tube stability. 2H-11 cells were placed on Matrigel and allowed to form tube-like networks. JAR cells transfected as in A were then plated on the networks, and total network area was measured at the times indicated. JAR cells expressing DR-ID2 destabilize 2H-11 cell networks at 16 hours, while cells expressing ASB4 or ASB4 and wild-type ID2 maintained the size of these 2H-11 cell networks compared to vector transfected cells at 48 hours after plating (D).* p<0.05, *** p<0.01 as compared to vector/vector.

In culture, TB cells can induce endothelial turnover [37] and increase the stability of endothelial cell networks [38], recapitulating the *in vivo* events that occur when TB cells are differentiating into endothelial-like cells. Because *Asb4^−/−^* placentas express markers of undifferentiated TB cells (Figure 2), we hypothesized that ASB4 would increase the JAR cell-meditated apoptosis of 2H-11 endothelial cells as well as the vascular stability of vessel-like networks formed by 2H-11 cells in culture. We observed that JAR cells transfected with ASB4 induced 2H-11 endothelial cells to apoptose 3-fold more than vector control cells. Co-expression of wild-type ID2 did not attenuate apoptosis, but JAR cells co-expressing DR-ID2 and ASB4 resulted in significantly fewer TUNEL-positive 2H-11 cells compared with JAR cells that were only transfected with ASB4. Of note, DR-ID2 and ASB4 co-expression elevated apoptosis compared with vector control cells, but this was significantly less than cells that did express ASB4 alone (Figure 5A, B). These data demonstrate that ASB4 promotes a functional vascular phenotype that recapitulates *in vivo* endothelial replacement with differentiating TB cells and that ID2 represses this effect.

Previous reports demonstrated that endothelial cells induce TB migration in culture and that TB cells stabilize these endothelial vascular networks [38], representing a model of the *in vivo* events that occur during TB differentiation [41]. To examine whether ASB4 could promote TB cell stabilization of endothelial cell networks, we measured the ability of JAR cells transfected with *Asb4* and *Id2* to form stable vascular networks over time, using branching 2H-11 tube-like structures as the strata upon which JAR cells could migrate to and stabilize. In isolation, 2H-11 cells plated on Matrigel consistently form branching tube-like structures within approximately four hours [48] but devolve into spheroid cluster of cells within 16 hours (data not shown). However, the addition of trophoblast cells can stabilize these networks for days and even weeks in culture [38]. Therefore, we hypothesized that *Asb4*-transfected JAR cells would stabilize these 2H-11 cell networks for significantly longer than vector-transfected JAR control cells. JAR cells expressing ASB4, either alone or co-transfected with wild-type *Id2*, were able to maintain and stabilize the 2H-11 vascular networks well past 16 hours, when vector control cell networks had destabilized (Figure 5C, D). Cells co-transfected with *Asb4* and DR-*Id2* quickly and significantly destabilized 2H-11 networks at 16 hours and were indistinguishable from control networks after 48 hours. These results indicate that ASB4 promotes TB endothelial-like cell function *in vitro* and that ASB4 mediates these effects by degrading ID2, since DR-ID2 attenuates this ASB4-mediated effect in placental cells.

**Figure 6:**
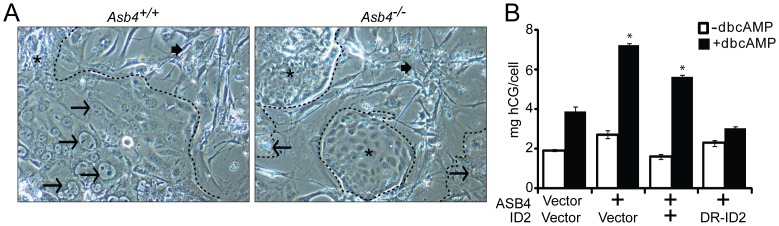
ASB4 promotes TB cell differentiation *in vitro*. A) TB stem cells (TBSCs) were isolated from wild-type and *Asb4^−/−^* extraembryonic ectoderm at E7.5. Cells isolated from each conceptus were cultured in isolation, and these data represent 4 unique populations of cells for each genotype. Serum withdrawal induces the formation of large, multinucleated trophoblast giant cells (TGCs, arrows) that differentiate from TBSCs (asterisks). As shown, wild-type TBSCs largely differentiate into TGCs (left panel) while *Asb4^−/−^* cells remain in undifferentiated embryoid bodies (right panel). MEF-feeder cells are indicated by filled arrows. Dashed outlines indicate the border of non-MEF cell clusters. B) JAR cells were transfected to express vector, *Asb4*, or *Asb4* co-transfected with vector, wild-type *Id2*, or degradation-resistant *Id2* (DR-*Id2*). ASB4 induced hCG secretion, and co-expression of wild-type ID2 with ASB4 did not change hCG secretion compared to ASB4 expression alone. DR-ID2 prevented dcbAMP-induced hGC section, with concentrations of hCG no different than vector/vector transfected cells. * p<0.01 compared with vector/vector.

Because ASB4 mediates vascular differentiation in ES cells [15] and we have demonstrated that ASB4 negatively regulated the anti-differentiation protein ID2 (Figure 4A, B), we hypothesized that ASB4 would mediate placental cell differentiation through the regulation of ID2 and tested this hypothesis *in vitro*. First, TBSCs were isolated from the extraembryonic ectoderm of early post-implantation (E7.5) wild-type and *Asb4^−/−^* embryos and cultured on a feeder layer of mitotically inactivated MEFs, which promote the long-term maintenance and proliferation of undifferentiated stem cells [15]. Large-scale multipotent differentiation is expected for the first several passages, so cultures were grown 6–8 weeks prior to serum-withdrawal. Terminally differentiated cells were sub-cultured out, leaving only the undifferentiated embryoid bodies of TBSCs. Although the factors required for TBSC-to-endothelial transformation are not yet know, TBSCs readily differentiate into trophoblast giant cells (TGCs) [35]. Based on previous work from this laboratory [49], we used serum withdrawal to promote TBSC differentiation. Thus, we used the appearance of TGCs as an index of TBSC differentiation. After serum withdrawal for 72 hours, we visualized the isolated cells with bright-field microscopy. As shown in Figure 6A, wild-type TBSCs largely differentiated into large, multinucleated TGCs, which were morphologically very different from the small, clustered, undifferentiated TBSCs that form embryoid bodies as seen in *Asb4^−/−^* cells (right panel). Further, differentiated TGCs laid flat on the culture dish, while undifferentiated embryoid bodies had raised edges and appeared more convex on the culture dish, allowing for easy identification.

To determine whether ASB4’s influence on TB cell differentiation involves ID2, we examined human chorionic gonadotropin (hCG) secretion, a well-established marker of trophoblast differentiation [36], in JAR cells that ectopically express ASB4 and ID2. hCG secretion was stimulated via the addition of dbcAMP to the growth medium following the indicated transfection for 48 hours and was subsequently measured in the medium by ELISA. ASB4 stimulated hCG production approximately 2 fold compared with the vector control. Co-transfecting wild-type *Id2* with ASB4 did not abolish hCG production, but co-transfection of *Asb4* and DR-*Id2* prevented hCG stimulation (Figure 6B). Together with data in Figure 5, these results illustrate that ASB4 promotes placental cell differentiation and function *in vitro,* and that ID2 mutants resistant to ASB4-mediated degradation can inhibit the differentiation and function of TB cells *in vitro.*

### Asb4^−/−^ Mice Phenocopy Human Patients with Pre-eclampsia

**Figure 7:**
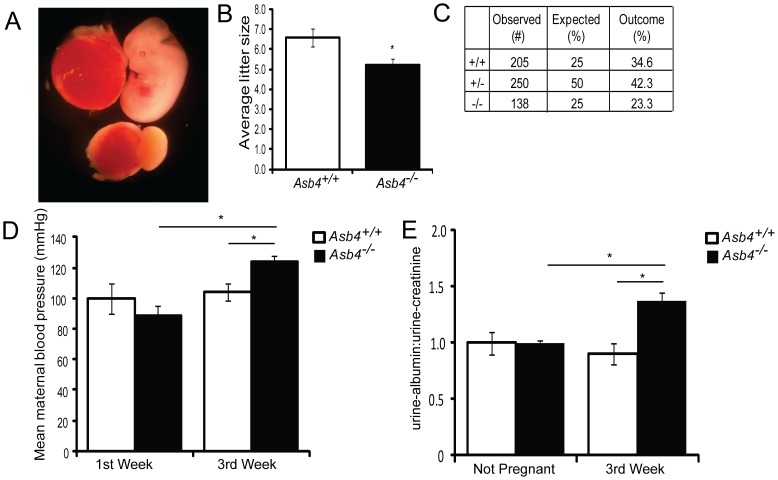
Pregnant *Asb4^−/−^* mice display symptoms of pre-eclampsia. A) A subset of *Asb4^−/−^* embryos dies *in utero*. *Asb4^−/−^* littermates are shown at E12.5, illustrating the lack of placental vasculature and dramatically reduced fetal growth in a subset of *Asb4^−/−^* embryos. The resultant average litter size, taken from more than 25 litters from each group, is quantified in B. C) Heterozygous breeding results in a lower than expected number of *Asb4^−/−^* pups (p<0.01, Fisher’s exact test). Pregnant *Asb4^−/−^* mice have significantly elevated mean blood pressure (D) and urine-albumin:urine-creatinine (E) in the third trimester of pregnancy compared with both *Asb4^−/−^* mice in the first week of pregnancy and wild-type mice in the third week of pregnancy. * p<0.01.

Because our data indicate that ASB4 mediates placental cell differentiation and function (Figures 4 and 5), and that *Asb4* deletion has negative consequences in the placental vasculature throughout development (Figure 2 and Figure S2), we investigated whether the placental abnormalities found in *Asb4^−/−^* mouse placentas contributed to the placenta-specific disease pre-eclampsia, whose pathogenesis may stem from abnormal placental vascular development [6]. *Asb4^−/−^* female mice produced significantly smaller litter sizes compared with wild-type female mice (Figure 7B) due to spontaneous abortion mid-gestation (Figure 7A). Similarly, *Asb4^+/−^* breeding pairs produced non-Mendelian ratios of pups that were significantly skewed toward higher numbers of wild-type animals at the expense of *Asb4^−/−^* pups (Figure 7C). When investigating the source of lethality in the *Asb4^−/−^* pregnancies, we observed that fetal growth halted at approximately E10.5 to E11.5 in a subset of *Asb4^−/−^* embryos. These embryos lacked functioning placental vascularization (Figure 7A, and data not shown), which may contribute to the abortion and fetal reabsorption seen in *Asb4^−/−^* embryos [4].

Because ID2 expression is elevated in trophoblast cells placentas of women with pre-eclampsia [25] and *Asb4^−/−^* mouse placentas (Figure 2D, E), combined with the vascular defects observed in *Asb4^−/−^* placentas (Figure 2A, Figure S2), we investigated whether our *Asb4^−/−^* mice shared traits with human patients with pre-eclampsia, which is widely believed to be a disease of the placental vasculature [14]. Two hallmarks of pre-eclampsia are maternal hypertension and proteinuria during late-stage pregnancy. Pregnant *Asb4^−/−^* female mice had increased blood pressure during late gestation (E14-term), as compared to both gestationally age-matched wild-type mice and *Asb4^−/−^* mice during the first week of gestation (Figure 7D). Further, pregnant *Asb4^−/−^* female mice had higher ratios of albumin:creatinine protein in their urine during late stage pregnancy than wild-type mice (Figure 7E). Together, these results suggest that *Asb4^−/−^* mice phenocopy human pre-eclampsia and may serve as a model for both early placental vascularization and human placental disease.

## Discussion

Strict control over the vascular patterning of the placenta is critical for both maternal and fetal survival [50]. Aberrant differentiation events early in development negatively affect the later formation of the vasculature [41], but relatively little is known what drives early differentiation events. Although none of the limited data that identify putative substrates or functions of ASB4 support a central function for ASB4 *in vivo* [15], [16], [22], [51], [52], prior work from this laboratory has shown that ASB4 is involved in early vascular differentiation and is highly expressed in the developing placenta [15]. Therefore, we utilized *Asb4^−/−^* mice, in conjunction with placenta-derived cells, to determine the function of ASB4 during placental vascular differentiation. Consistent with our previous work [15], we found that ASB4 is largely localized to the early endothelium in the placenta. We also found that *Asb4* deletion induces the expression of markers of undifferentiation in the placenta, including the anti-differentiation protein ID2. Based on this data, along with the expression pattern of various markers of TB cells and endothelial differentiation in *Asb4^−/−^* placentas, we determined that ASB4 is involved in the earlier stages of differentiation events, and the consequences of *Asb4* deletion persist into later stages of gestation resulting in insufficient placental vascularization.

Due to the limited information found in the literature, identifying a substrate of ASB4s ligase activity was central to this investigation. Taking a candidate approach, we reasoned that any ASB4 substrate would have to share its narrow spatiotemporal expression pattern, contribute to vascular phenotypes, and be involved in differentiation. We determined that the ID family of proteins would fulfill these criteria [53]. The ID proteins (ID1 to ID4) are known to mediate differentiation and cell cycle control, which impact cell functions such as metastasis, angiogenesis, apoptosis, and maintaining stemness [30], [53]–[55]. Within these processes, there is significant, but not ubiquitous redundancy between the individual ID proteins [56]–[59]. Further, ID proteins, and ID2 specifically, are known to be tightly regulated by E3 ligases [47], [60], [61]. Therefore, ID2 was chosen for investigation in this study due to its involvement in differentiation [54], vascular development [62], and placental maturation [25]. Using cell culture and biochemical techniques, we determined that ASB4 can directly negatively regulate ID2 expression.

Placental remodeling requires three unique vascular events for proper function: TBSC differentiation, replacement of endothelium with trophoblast cells, and vascular stabilization to form high capacity vessels [41]. Possibly because the JAR cell line was isolated from CTB cells in choriocarcinomas, these cells can be induced to mimic *in vivo* cells under certain conditions. We adapted several methods to assess cell differentiation and function in culture, and whether ASB4 promoted these events through the inhibition of ID2. Although these methods do not completely recapitulate *in vivo* events, they collectively indicate that ASB4 has a pro-vascular differentiation function in placental cells. By ectopically expressing ASB4 in the JAR cells, we were able to determine that ASB4 promotes all three aspects of placental vascular remodeling. In addition, using isolated TBSCs, we were able to observe primary TB cell differentiation in culture. TBSCs that lack *Asb4* remained in undifferentiated embryoid bodies, in contrast to wild-type TBSCs which differentiated into TGCs upon serum withdrawal. Because exact markers of TBSC and endothelial differentiation are not well defined in the placenta, future studies will be needed to more precisely address these differentiation events.

Using *Asb4^−/−^* mice as a model for ASB4 function *in vivo*, we explored the phenotypic consequences of *Asb4* deletion, focusing particularly on the early placenta. Although the majority of *Asb4^−/−^* embryos survived to term, all had placentas with varying degrees of vascular dysfunction. Further, embryonic lethality occurred in a subset of *Asb4^−/−^* embryos at approximately E10.5 due to gross endothelial disruption in the placenta. Furthermore, placental vascular dysfunction in *Asb4^−/−^* placentas also had deleterious effects on pregnant mice, phenocopying women with pre-eclampsia. Though extremely common, little is known about the pathogenesis of pre-eclampsia [63] and to date there is no cure other than delivery of the placenta. Both third trimester hypertension and proteinuria, hallmarks of pre-eclampsia, were recapitulated in *Asb4^−/−^* mothers. This disease state, in conjunction with the differentiation defects in the placenta of *Asb4^−/−^* mice, provides a unique model of early vascular development. Ultimately, this model of pre-eclampsia and vascular dysfunction may be used to investigate therapeutic strategies for treating pre-eclampsia and other diseases of the placenta.

## Supporting Information

Figure S1 **Diminished mature vasculature in** ***Asb4^−/−^*** **placentas is not due to increased apoptosis or abnormal proliferation.** E15.5 placental sections from wild-type and *Asb4^−/−^* mice were evaluated for aberrant proliferation or apoptosis using phospho-histone H3 (pH3) or TUNEL, respectively. In both cases, no discernible differences were noted between genotypes. BV; blood vessel. Arrows denote TUNEL-positive cells.
(TIF)

Figure S2 ***Asb4*** **deletion induces vascular dysfunction and mislocalization of blood vessels in the placenta.** A) Near-term (E17.5) placental sections were harvested and labeled with von Willibrand factor to measure thrombus response and DBA to determine uterine natural killer cell response. *Asb4^−/−^* placentas display elevated thrombus/thrombosis response (left panel) compared with wild-type placentas, indicating damaged vasculature. Further, there is a dramatic increase in activated uterine natural killer cells (right panel) in *Asb4^−/−^* tissues, indicating elevated macrophage and immune response, compared to wild-type tissue. B) E17.5 placental sections were stained with hematoxylin and eosin and examined for gross morphology. Blood vessels (arrows) were counted and classified based on their location in the labyrinth (LZ), junctional (JZ), or decidual (DE) zones. Blood vessels in wild-type placentas are seen at the edge of the deciduas in, whereas significantly more vessels in *Asb4^−/−^* placentas are located in the junctional zone, at the expense of the decidual zone, which is quantified in C, indicating that vascular invasion/migration is defective in the absence of *Asb4*. * p<0.01 compared to wild-type.
(TIF)

Figure S3 **N-terminally tagged ID2 is resistant to ASB4-mediated degradation.** A) JAR cells were transfected with wild-type *Id2*, *Id2* lacking all lysine residues (LL-*Id2*), or *Id2* with 6xMyc tags on either the N-terminus (DR-*Id2*) or the C-terminus (DS-*Id2*) in the absence or presence of ASB4. ASB4 is unable to degrade DR-ID2 but can efficiently degrade other ID2 mutants, which is quantified in B. * p<0.01 compared to wild-type ID2.
(TIF)

Figure S4 **ASB4 degrades ID2 in a proteasome-dependant manner, and does not affect ID2 cellular location.** A) JAR cells co-transfected with *Id2* and either vector or wild-type *Asb4* were treated with DMSO or MG-132. While overall ID2 expression increases in the presence of MG-132, ID2 expression decreases only in the presence of ASB4 in DMSO-treated cells, suggesting that ID2 is sensitive to proteasomal degradation when co-expressed with ASB4. B) JAR cells were transfected as in A, then treated with cycloheximide for the indicated times. In the presence of ASB4 (right panel above, dashed line and open diamonds in graph), ID2 half-life is shortened from 40.2 minutes to 33 minutes compared to cells that only express ID2 (left panel above, solid line and solid boxes in graph) indicating that ASB4 mediates ID2 protein expression. C) ID2 sub-cellular localization is not altered in the presence of ASB4. JAR cells transfected with *Id2* and either vector or wild-type *Asb4* were fractionated into the whole cell lysate (WCL), cytoplasmic (Cyto), nuclear (Nuc), and Triton-insoluble pellet (Pel) fractions. In all fractions, ID2 expression decreases in the presence of ASB4.
(TIF)
