# Supplementary figures and images for: The Ubiquitin Ligase ASB4 Promotes Trophoblast Differentiation through the Degradation of ID2
Source: PLoS One. 2014 Feb 21;9(2):e89451. doi: 10.1371/journal.pone.0089451 (PMC3931756; doi:10.1371/journal.pone.0089451)

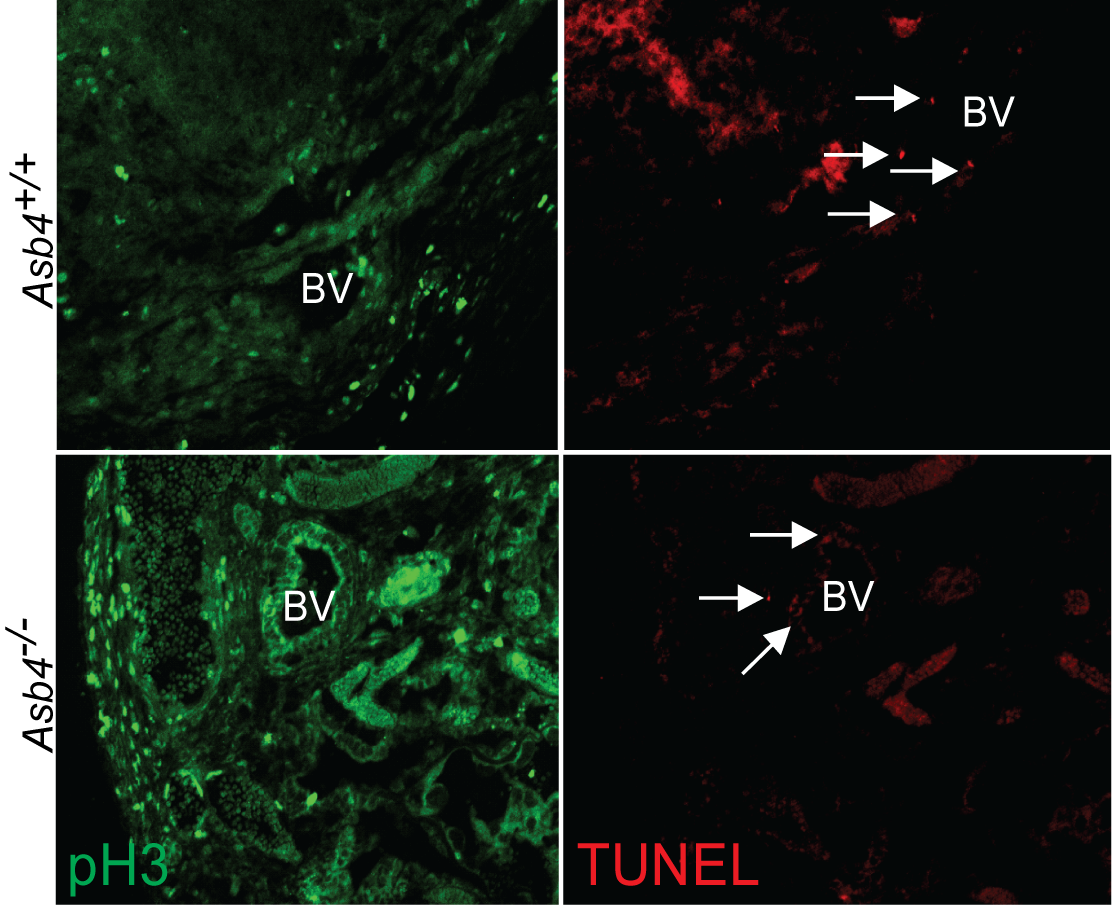

Supplement: Figure S1 — Diminished mature vasculature in Asb4−/− placentas is not due to increased apoptosis or abnormal proliferation. E15.5 placental sections from wild-type and Asb4−/− mice were evaluated for aberrant proliferation or apoptosis using phospho-histone H3 (pH3) or TUNEL, respectively. In both cases, no discernible differences were noted between genotypes. BV; blood vessel. Arrows denote TUNEL-positive cells. (TIF) [file pone.0089451.s001.tif]

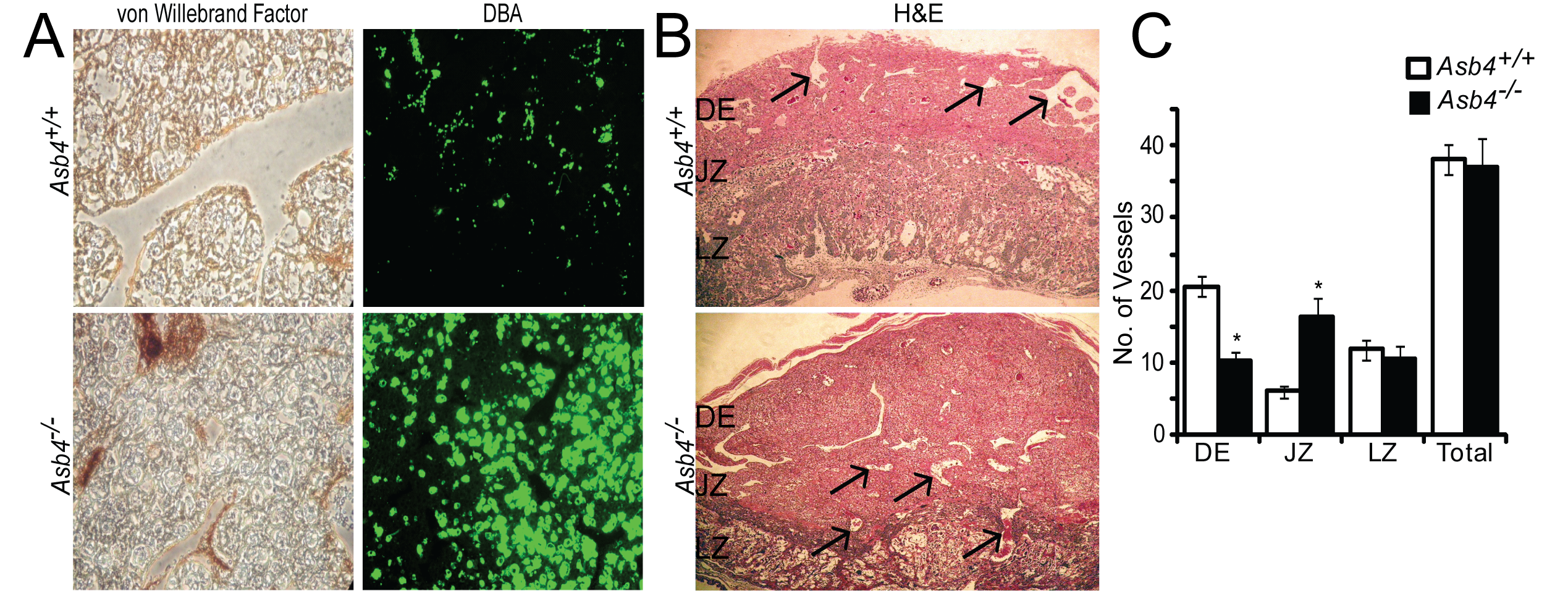

Supplement: Figure S2 — Asb4 deletion induces vascular dysfunction and mislocalization of blood vessels in the placenta. A) Near-term (E17.5) placental sections were harvested and labeled with von Willibrand factor to measure thrombus response and DBA to determine uterine natural killer cell response. Asb4−/− placentas display elevated thrombus/thrombosis response (left panel) compared with wild-type placentas, indicating damaged vasculature. Further, there is a dramatic increase in activated uterine natural killer cells (right panel) in Asb4−/− tissues, indicating elevated macrophage and immune response, compared to wild-type tissue. B) E17.5 placental sections were stained with hematoxylin and eosin and examined for gross morphology. Blood vessels (arrows) were counted and classified based on their location in the labyrinth (LZ), junctional (JZ), or decidual (DE) zones. Blood vessels in wild-type placentas are seen at the edge of the deciduas in, whereas significantly more vessels in Asb4−/− placentas are located in the junctional zone, at the expense of the decidual zone, which is quantified in C, indicating that vascular invasion/migration is defective in the absence of Asb4. * p<0.01 compared to wild-type. (TIF) [file pone.0089451.s002.tif]

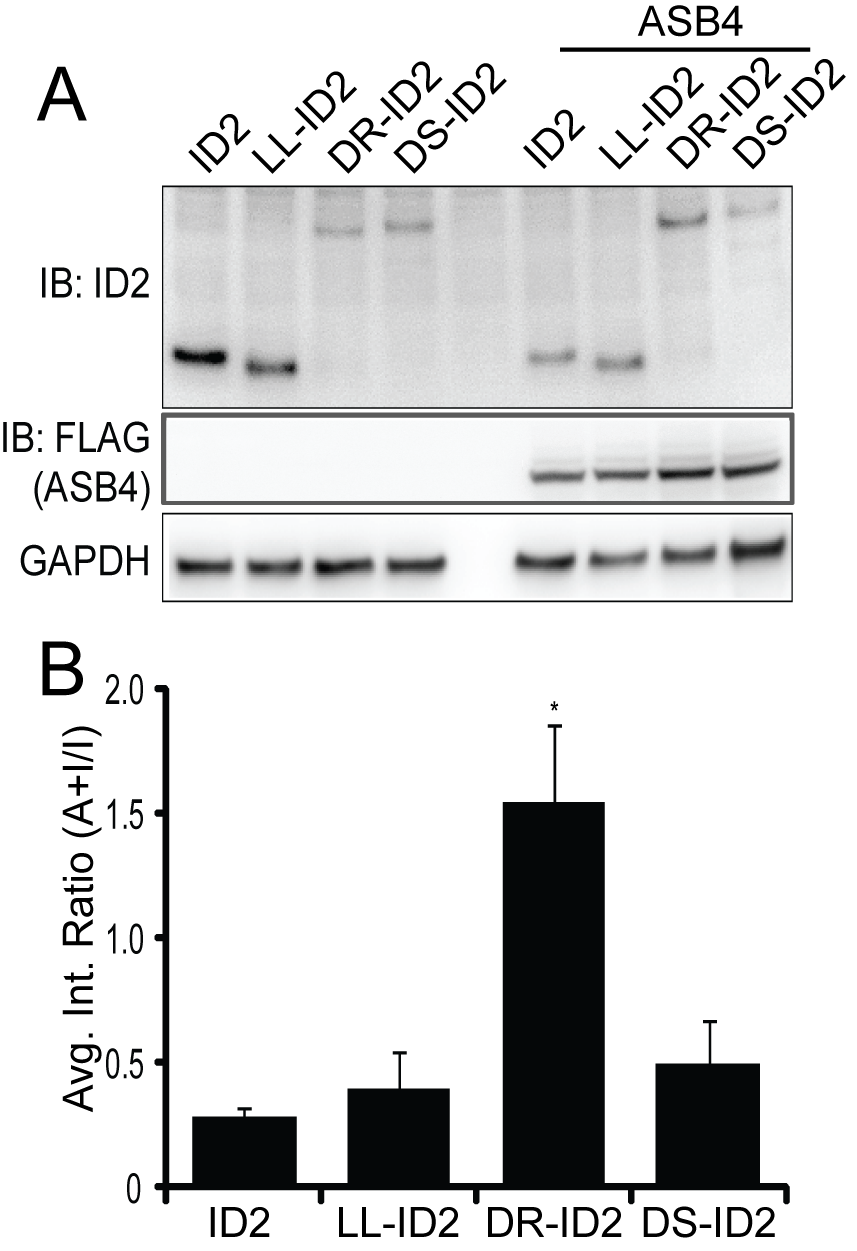

Supplement: Figure S3 — N-terminally tagged ID2 is resistant to ASB4-mediated degradation. A) JAR cells were transfected with wild-type Id2, Id2 lacking all lysine residues (LL-Id2), or Id2 with 6xMyc tags on either the N-terminus (DR-Id2) or the C-terminus (DS-Id2) in the absence or presence of ASB4. ASB4 is unable to degrade DR-ID2 but can efficiently degrade other ID2 mutants, which is quantified in B. * p<0.01 compared to wild-type ID2. (TIF) [file pone.0089451.s003.tif]

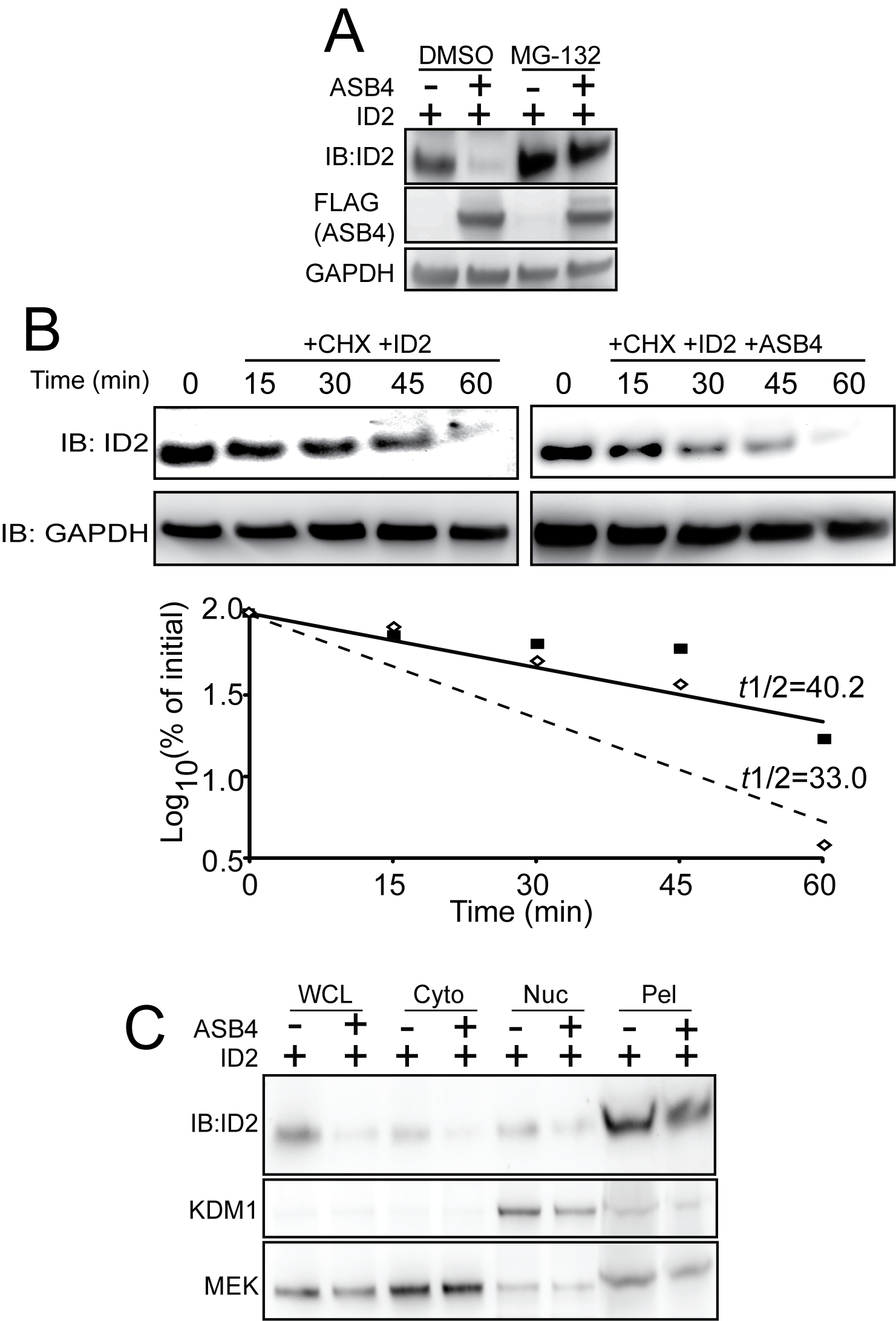

Supplement: Figure S4 — ASB4 degrades ID2 in a proteasome-dependant manner, and does not affect ID2 cellular location. A) JAR cells co-transfected with Id2 and either vector or wild-type Asb4 were treated with DMSO or MG-132. While overall ID2 expression increases in the presence of MG-132, ID2 expression decreases only in the presence of ASB4 in DMSO-treated cells, suggesting that ID2 is sensitive to proteasomal degradation when co-expressed with ASB4. B) JAR cells were transfected as in A, then treated with cycloheximide for the indicated times. In the presence of ASB4 (right panel above, dashed line and open diamonds in graph), ID2 half-life is shortened from 40.2 minutes to 33 minutes compared to cells that only express ID2 (left panel above, solid line and solid boxes in graph) indicating that ASB4 mediates ID2 protein expression. C) ID2 sub-cellular localization is not altered in the presence of ASB4. JAR cells transfected with Id2 and either vector or wild-type Asb4 were fractionated into the whole cell lysate (WCL), cytoplasmic (Cyto), nuclear (Nuc), and Triton-insoluble pellet (Pel) fractions. In all fractions, ID2 expression decreases in the presence of ASB4. (TIF) [file pone.0089451.s004.tif]
